# Effects of Nutritional Status During Sexual Maturation and Resource Availability on the Resource Allocation of Females in Burying Beetles

**DOI:** 10.1002/ece3.70808

**Published:** 2025-01-11

**Authors:** Wenxia Wang, Guojun Zhou, Wei Zhang, Kai Tian, Lunguang Yao

**Affiliations:** ^1^ Henan Field Observation and Research Station of Headwork Wetland Ecosystem of the Central Route of South‐To‐North Water Diversion Project College of Life Sciences, Nanyang Normal University Nanyang China; ^2^ Nanyang Medical College Nanyang China; ^3^ School of Life Science, Zhengzhou Normal University Zhengzhou China

**Keywords:** life‐history trade‐offs, *Nicrophorus vespilloides*, nutritional status, resource allocation, resource availability, sexual maturation

## Abstract

Resource availability should have consequences for life‐history functions and trade‐offs among them because it influences the amounts of resources allocated to different functions. Nutritional status during a key developmental window (sexual maturation) may also have an important impact on life‐history functions and such trade‐offs. However, less is known about whether and how they interact to influence the resource allocation of individuals. Here, we simultaneously manipulated female nutritional status during sexual maturation and resource availability during breeding in a burying beetle *Nicrophorus vespilloides* . We then monitored the main and interactive effects of these two factors on somatic maintenance and reproductive performance of burying beetle females. We found that variation in nutritional status during sexual maturation affects the resource allocation of burying beetle females only at the pre‐hatching stage. Poor‐fed females compensated for the initial differences in energy reserves by feeding from the carcass or engaged in terminal investment strategy and invested heavily at the post‐hatching stage. Specifically, poor‐fed females allocated more into somatic maintenance (gained more weight) and less into reproduction (provided less pre‐hatching care) than well‐fed females, whereas they provided a similar amount and duration of post‐hatching care. In addition, burying beetles with different nutritional statuses vary in their response to resource availability. Poor‐fed females allocated more into both somatic maintenance (gained more weight) and reproduction (provided more pre‐hatching care) when bred on large versus small carcasses, whereas well‐fed females tend to work near their maximum capacity and thus show no response to resource availability. Finally, our findings suggest that poor‐fed females did not suffer a future cost in offspring performance. Meanwhile, a large carcass allowed females to produce more and heavier offspring. These findings enhance our understanding of how important nutritional status during a key developmental window and resource availability during breeding is for the expression of resource allocation.

## Graphical abstract

Variation in nutritional status during sexual maturation affects the resource allocation of burying beetle females only at the pre‐hatching stage; the long‐term effects may diminish with time as females compensate for the differences in their energy reserves by feeding from the carcass or engage in terminal investment strategy at the post‐hatching stage. Moreover, burying beetles with different nutritional statuses vary in their response to resource availability during breeding. Poor‐fed females allocate more into both self‐maintenance and reproduction as carcass size increased, whereas well‐fed females show no response to carcass size.

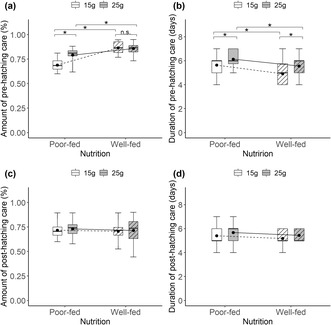

## 1 Introduction

Individuals must allocate resources between different life‐history functions such as somatic maintenance and reproduction, current and future reproduction, and offspring number and size (Williams 1966; Van Noordwijk and de Jong 1986; Stearns 1989). It is because these functions compete for the same pool of limited resources and increased allocation to one function will lead to reduced allocation to the other (Van Noordwijk and de Jong 1986). Therefore, resource availability should have an important impact on life‐history functions and trade‐offs given that such variation determines how many resources can be allocated to different functions (Van Noordwijk and de Jong 1986; Stearns 1989). For iteroparous organisms, there are two general patterns of resource allocation strategies that have been hypothesized (i.e., terminal investment strategy and reproductive restraint strategy). The terminal investment strategy proposes that individuals should increase investment in the current reproduction to maximize the lifetime reproductive output when their residual reproductive value decreases (Williams 1966). In contrast, the reproductive restraint strategy predicts that individuals should decrease investment in the current reproduction to reserve more resources for future reproductive opportunities (Clutton‐Brock 1984; McNamara et al. 2009). Both strategies predict changes in resource allocation between current and future reproduction; individuals can facultatively switch from reproductive restraint to terminal investment when their status deteriorates below a certain threshold value (Duffield et al. 2017). Previous studies have tested how individuals adjust their resource allocation based on resource availability by manipulating food acquisition and then investigating the negative relationships between different life‐history functions (e.g., offspring number vs. size) (Smiseth et al. 2014; Richardson and Smiseth 2020) or then detecting costs of current reproduction on future reproduction (Lambert and Smiseth 2022; Wang et al. 2022). It has been suggested that large amounts of resources may mitigate the negative relationships or even lead to positive correlations among different functions and thus masking the underlying life‐history trade‐offs, whereas limited resources may intensify such trade‐offs (Zera and Harshman 2001; King, Roff, and Fairbairn 2011; Smiseth et al. 2014; Descamps et al. 2016). However, individuals may adjust their resource allocation based on both extrinsic and intrinsic factors that together influence the residual reproductive value.

Variation in nutritional status is an important intrinsic aspect of individuals that may influence their resource allocation. Several studies have revealed how nutritional status prior to breeding may influence life‐history functions and trade‐offs among them by manipulating the nutritional conditions and then comparing the response of well‐fed and poor‐fed individuals (Clark, Zera, and Behmer 2015; Krause, Krüger, and Pogány 2017; Holden et al. 2019; Keppner et al. 2023; Lambert and Smiseth 2024). It has been suggested that poor nutritional status generally results in reduced allocation to reproduction (e.g., reduced fecundity, lower parental investment) (Nagy and Holmes 2005; Warner, Lovern, and Shine 2007; Salomon et al. 2011; Segers, Gerber, and Taborsky 2011; Wong and Kölliker 2012). In contrast, good nutritional status may confer lasting advantages to individuals and mask the expression of life‐history trade‐offs to some degree (Schluter, Price, and Rowe 1991; Blanckenhorn and Heyland 2005). Furthermore, individuals may be particularly sensitive to their nutritional status when undergoing key developmental stages (sexual maturation) because it may influence their physiology, morphology, and behavior in adulthood (Metcalfe and Monaghan 2001; Barrett et al. 2009; Barrett, Moore, and Moore 2009; Hopwood, Moore, and Royle 2013; Wilner et al. 2020). Individuals may therefore follow different resource allocation strategies and express alternative life‐history trajectories (Metcalfe and Monaghan 2001; Taborsky 2006). In addition, the effects of nutritional status during sexual maturation may also interact with the effects of resource availability during breeding. If resource availability becomes more favorable during the subsequent breeding, individuals may compensate for a bad start (Ali, Nicieza, and Wootton 2003). Although we have a good understanding of the impacts of nutritional status prior to breeding on the resource allocation of individuals, it is unclear whether nutritional status during sexual maturation can have different effects. Moreover, it remains unclear whether they can interact with the effects of resource availability during the subsequent breeding. In this study, we simultaneously manipulated nutritional status during sexual maturation and resource availability during breeding to assess their separate and interactive effects on the resource allocation in the burying beetle ( *Nicrophorus vespilloides* ).

Burying beetles (*Nicrophorus* spp.) breed on carcasses of small vertebrates, which serve as the sole food resource for both parents and their developing offspring until larvae dispersal (Eggert, Reinking, and Müller 1998; Scott 1998). Parents can adjust offspring number based on carcass size through filial cannibalism (Bartlett 1987). Burying beetle parents also provide complex and flexible pre‐hatching and post‐hatching care for their offspring (Eggert, Reinking, and Müller 1998). Specifically, the pre‐hatching care consists of preparing and maintaining the carcass by removing fur (hair), rolling it into a ball, burying it underground, and applying antimicrobial secretions to prevent decomposition (Eggert, Reinking, and Müller 1998; Rozen, Engelmoer, and Smiseth 2008; Trumbo 2017). The post‐hatching care mainly includes guarding their offspring against intruders and provisioning them with pre‐digested carcasses (Eggert, Reinking, and Müller 1998; Smiseth and Moore 2002; Potticary et al. 2024). Although both parents are competent in all activities, males typically spend more time on the pre‐hatching care, whereas females are more involved in the post‐hatching care and stay on the carcass for longer than males until larvae dispersal (Eggert, Reinking, and Müller 1998; Smiseth and Moore 2004; Ratz et al. 2021). After hatching, larvae obtain food by self‐feeding directly inside the carcass or begging for pre‐digested carcasses from their parents (Eggert, Reinking, and Müller 1998; Smiseth and Moore 2002). Larvae do not feed once they leave the carcass until eclosion, and thus their adult body size is determined by larvae size at dispersal (Bartlett and Ashworth 1988). After eclosion, subadults must feed for several days (approximately 10 days) to attain sexual maturation (Wilson and Knollenberg 1984; Trumbo, Borst, and Robinson 1995). It has been suggested that there are trade‐offs between offspring number and size only when they breed on small carcasses (Smiseth et al. 2014; Wang et al. 2022). Previous studies have demonstrated that burying beetles in poor nutritional status prior to breeding consume more from the carcass to enhance their energy reserves but produce fewer and smaller offspring than beetles in good nutritional status (Gray et al. 2018; Keppner, Ayasse, and Steiger 2018). Burying beetles that experienced poor nutritional status during sexual maturation are much less successful in competition for carcasses (Hopwood, Moore, and Royle 2013). However, the influences of nutritional status during sexual maturation on the resource allocation are still largely unknown. Furthermore, it remains unknown whether individuals can compensate for a poor start in life (i.e., poor nutritional status during sexual maturation) by reserving more energy when resource availability becomes more favorable during the subsequent breeding. That is, it remains unclear whether there is an interactive effect between nutritional status during sexual maturation and resource availability during breeding on the resource allocation in burying beetles.

In this study, we aimed to examine how burying beetle females adjust their resource allocation based on nutritional status during sexual maturation, resource availability during breeding, and their interactive effects. To this end, we used a two‐by‐two factorial design in which we simultaneously manipulated females’ nutritional status during sexual maturation (by providing them with different kinds of food supply: mealworms vs. carcasses) and resource availability during breeding (by providing them with different amounts of resource: small vs. large carcasses). We then assessed the effects of such manipulations on subsequent life‐history functions associated with somatic maintenance (weight change), reproductive investment (the amount and duration of pre‐hatching/post‐hatching care), and reproductive output (egg‐laying time, larvae number, average larval mass). We predicted that poor‐fed females would allocate more into self‐maintenance rather than reproduction to maintain their basic physiological processes because they may lack enough energy to meet both somatic and reproductive requirements (Keppner, Ayasse, and Steiger 2018). We also predicted that poor‐fed females would produce fewer offspring and provide less care than well‐fed females because they may take longer to complete carcass preparation and thus have more chance to feed from the carcass, in turn reducing the resource availability for their offspring (Keppner, Ayasse, and Steiger 2018). In addition, we predicted that the effects of resource availability would be more pronounced for poor‐fed females than for well‐fed females. Poor‐fed and well‐fed females would respond differently to carcass size. With increasing carcass size, poor‐fed females would have more chance to consume from the carcass, produce more offspring, and provide more care, whereas well‐fed females would show no response in their weight gain and parental care. This is because poor‐fed females are expected to replenish their energy reserves by feeding from the carcass and larger carcasses give them a chance to invest more into both self‐maintenance and reproduction (Smiseth et al. 2014; Gray et al. 2018; Keppner, Ayasse, and Steiger 2018). However, well‐fed females may have enough energy reserves and thus typically work to their physical limitations regardless of carcass size.

## 2 Materials and Methods

### 2.1 Study Animals

All individuals used in this study were second‐generation laboratory‐reared offspring of burying beetles ( *N. vespilloides* ) collected in the Baotianman National Nature Reserve, Neixiang, China. All individuals were maintained at the College of Life Science in Nanyang Normal University and were kept under a 16:8‐h light: dark diurnal rhythm at 24° ± 2°C. To avoid inbreeding and ensure virginity before the experiment, up to five same‐sex subadult beetles that descended from the same family post‐eclosion were kept in plastic boxes (length: 20 cm; width: 15 cm; height: 10 cm) filled with 5‐cm moist soil.

### 2.2 Experimental Procedures

To manipulate nutritional status during sexual maturation, we placed subadult females individually in breeding boxes filled with 1‐cm moist soil and provided half of them with mealworms ( *Tenebrio molitor* ) twice 1 week with 2–3 mealworms per beetle each time (the same as all other laboratory‐reared individuals) for 2 weeks post‐eclosion. The other half was fed with 3–5‐g thawed mouse carcasses ( *Mus musculus* ). We chose these nutritional treatments because mealworms meet their developmental needs based on previous studies (Wang et al. 2021, 2022), and carcasses are superior resources for burying beetles (Scott 1998). Moreover, we chose 2 weeks as the treatment duration to ensure that poor‐fed females attain their sexual maturation. We then paired all females with unrelated virgin males randomly and kept them undisturbed in plastic boxes for 3 days to ensure that all females received enough sperm. After mating, we removed all males to exclude the potential effects of sexual conflict over parental care and carcass consumption (Pilakouta, Richardson, and Smiseth 2016; Smiseth 2019). We then provided females with a 15‐g or 25‐g carcass and checked whether they were present on or inside the carcass, or whether they were invisible in the soil three times daily (07:00–08:00 am, 13:00–14:00 pm, 19:00–20:00 pm, 5‐h intervals) by visual inspection until larvae dispersal. In burying beetles, presence on (preparing and maintaining the carcass) or inside (provisioning larvae) the carcass is a strong indicator of parental care (Walling et al. 2008; Head et al. 2014). Prior works also suggest that female burying beetles regurgitate most of the carcasses they consume to their offspring and that carcass consumption is a form of parental care (Walling et al. 2008; Mattey and Smiseth 2015; Andrews, Kruuk, and Smiseth 2016; Pilakouta, Richardson, and Smiseth 2016). Therefore, we estimated the amount of pre‐hatching/post‐hatching care as the proportion of times that females were present on or inside the carcass of the pre‐hatching/post‐hatching observation times. We calculated the duration of pre‐hatching care as the days from carcasses were provided to females until larvae hatching and the duration of post‐hatching as the days from larvae hatching until females were absent from the carcass for three consecutive observation times or larvae dispersed from the carcass (Benowitz et al. 2013; Head et al. 2014). To identify the time of oviposition, we checked for eggs three times daily (as described above) from the bottom of the breeding boxes (Monteith, Andrews, and Smiseth 2012). We then recorded the egg‐laying time as the number of days since females received the carcass until eggs were visible at the bottom of breeding boxes. After the larvae dispersed from the carcass, we recorded the larvae number and the total brood mass (accuracy: 0.01 g), and then calculated the average larval mass as the total brood mass divided by the larvae number. We also measured the body size (pronotum width, accuracy: 0.01 mm) of all parents because burying beetles adjust their parental investment and offspring performance based on their own and partner's body size (Steiger 2013; Pilakouta, Richardson, and Smiseth 2015). We weighted (accuracy: 0.0001 g) each female three times during the experiment: at the time of post‐eclosion (to exclude the potential effects of initial body weight), at the time of mating (to verify whether females differ in their nutritional status), and at the time of terminating care (to calculate their total weight change during breeding).

### 2.3 Statistics

All analyses were performed using R version 4.0.3 (R Core Team 2020). We used linear models (LMs) for traits that were continuous and had a normal structure (initial weight, weight change, average larval mass), generalized (mixed) linear models for traits that had a binomial error distribution (GLMMs: the amount of pre‐hatching and post‐hatching care), or Poisson error distribution (GLMs: larvae number, GLMMs: the duration of pre‐hatching and post‐hatching care, egg‐laying time). All models included the fixed factors of female nutritional status during sexual maturation (mealworms vs. carcasses) and carcass size during breeding (small vs. large) and their interactive effects. Group identity was included as a random factor in GL(M)Ms. When analyzing the post‐hatching care, we included the larvae number as a covariate because females may adjust their parental investment based on their brood size (Wang et al. 2021; Sahm et al. 2023). The treatment–covariate interactions were not significant and thus excluded in the final model. When analyzing the average larval mass, we originally included the larvae number as a covariate because the average larval mass may decrease with increasing brood size (Richardson and Smiseth 2020). The treatment–covariate interactions were highly significant, indicating that the homogeneity of the slope assumption was violated. The relationship between offspring size and number differs between different resource availability treatments; it was inappropriate to proceed with testing the significance of the fixed effects assuming a common slope. Therefore, we reported the results of the model of average larval mass excluding larvae number as a covariate; it did not change the significance of the fixed factors. We used the *anova* function to obtain Chi‐square and *p* values and post hoc Tukey contrasts to test differences whenever the interactive effects had significant impacts on the variables.

## 3 Results

**FIGURE 1:**
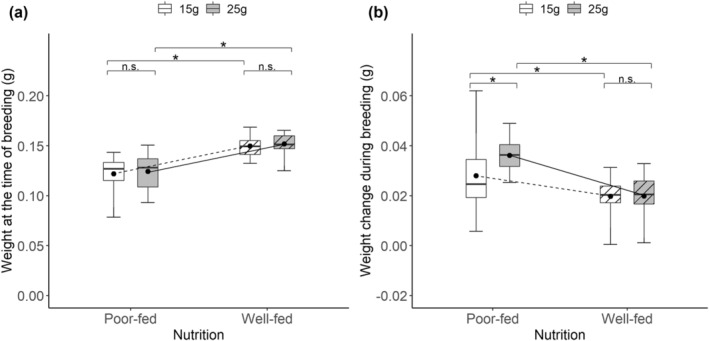
The differences in the body weight of females among different treatments at the time of breeding (a), and effects of nutritional status during sexual maturation (poor‐fed vs. well‐fed), carcass size during breeding (15 g vs. 25 g), and the interactive effects on the weight change of females during breeding (b). Black points on each box are mean values. The lines indicate the differences in mean values between different treatments. *Significant (*p* < 0.05); n.s., not significant.

The pronotum width and initial body weight of parents post‐eclosion did not differ among different treatments (Table 1); we thus excluded their potential effects on the resource allocation of individuals. In addition, the body weight of well‐fed females was significantly higher than poor‐fed females at the time of mating (*F* = 96.168, *p* < 0.001, Figure 1a); we thus confirmed that the nutritional treatments during sexual maturation had the intended effect of manipulating female nutritional status.

**TABLE 1:** Summary of three‐way ANOVA for pronotum width and initial body weight post‐eclosion of burying beetle parents among different nutritional statuses during sexual maturation (poor‐fed vs. well‐fed), carcass size during breeding (15 g vs. 25 g), and sex (male vs. female).

Explanatory variable	Pronotum width				Initial body weight			
	SS	MS	*F*	*p*	SS	MS	*F*	*p*
Nutritional status	0.15	0.151	0.443	0.51	0.001	0.001	1.060	0.305
Carcass size	0.07	0.067	0.197	0.66	0.000	0.000	0.016	0.898
Sex	0.03	0.028	0.083	0.77	0.001	0.001	0.866	0.353

*Note:* Poor‐fed treatments (15 g, *N* = 25; 25 g, *N* = 24); well‐fed treatments (15 g, *N* = 22; 25 g, *N* = 27).

Abbreviations: MS, mean square; SSs, sums of squares.

### 3.1 The Effects of Nutritional Status During Sexual Maturation and Resource Availability During Breeding on the Weight Change of Females

We found that there was a significant interactive effect on the weight change of females during the entire breeding (Table 2, Figure 1b). The pairwise comparisons showed that poor‐fed females gained more weight when bred on large versus small carcasses (Est = −0.008, *t* = −2.978, *p* = 0.019), whereas well‐fed females gained the same weight regardless of carcass size (Est = −0.001, *t* = −0.041, *p* = 0.99). In addition, poor‐fed females consumed more from the carcass and gained more weight than well‐fed females (Figure 1b).

**TABLE 2:** Effects of nutritional status during sexual maturation (poor‐fed vs. well‐fed), carcass size during breeding (15 g vs. 25 g), and the interactive effects on the weight change of females, the amount of pre‐hatching and post‐hatching care, the duration of pre‐hatching and post‐hatching care, and egg‐laying time, larvae number, average larval mass at dispersal.

Explanatory variable	Weight change		Amount of pre‐hatching care		Duration of pre‐hatching care		Amount of post‐hatching care		Duration of post‐hatching care		Egg‐laying time		Larvae number		Average larval mass	
	*F*	*p*	*χ* ^*2*^	*p*	*χ* ^*2*^	*p*	*χ* ^*2*^	*p*	*χ* ^*2*^	*p*	*χ* ^*2*^	*p*	*χ* ^*2*^	*p*	*F*	*p*
Nutrition status	40.78	**< 0.001**	35.81	**< 0.001**	14.94	**< 0.001**	0.36	0.55	2.71	0.09	19.12	**< 0.001**	0.01	0.96	0.09	0.76
Carcass size	4.58	**0.035**	7.00	**0.008**	11.80	**< 0.001**	0.31	0.58	3.42	0.06	29.15	**< 0.001**	6.23	**0.013**	4.80	**0.031**
Interaction	4.29	**0.041**	4.71	**0.030**	0.45	0.50	0.02	0.89	0.01	0.96	2.04	0.15	0.37	0.54	0.32	0.58

*Note:* Degrees of freedom are all equal to 1; significant *p*‐values are indicated in bold. Poor‐fed treatments (15 g, *N* = 25; 25 g, *N* = 24); well‐fed treatments (15 g, *N* = 22; 25 g, *N* = 27).

### 3.2 The Effects of Nutritional Status During Sexual Maturation and Resource Availability During Breeding on the Reproductive Investment of Females

**FIGURE 2:**
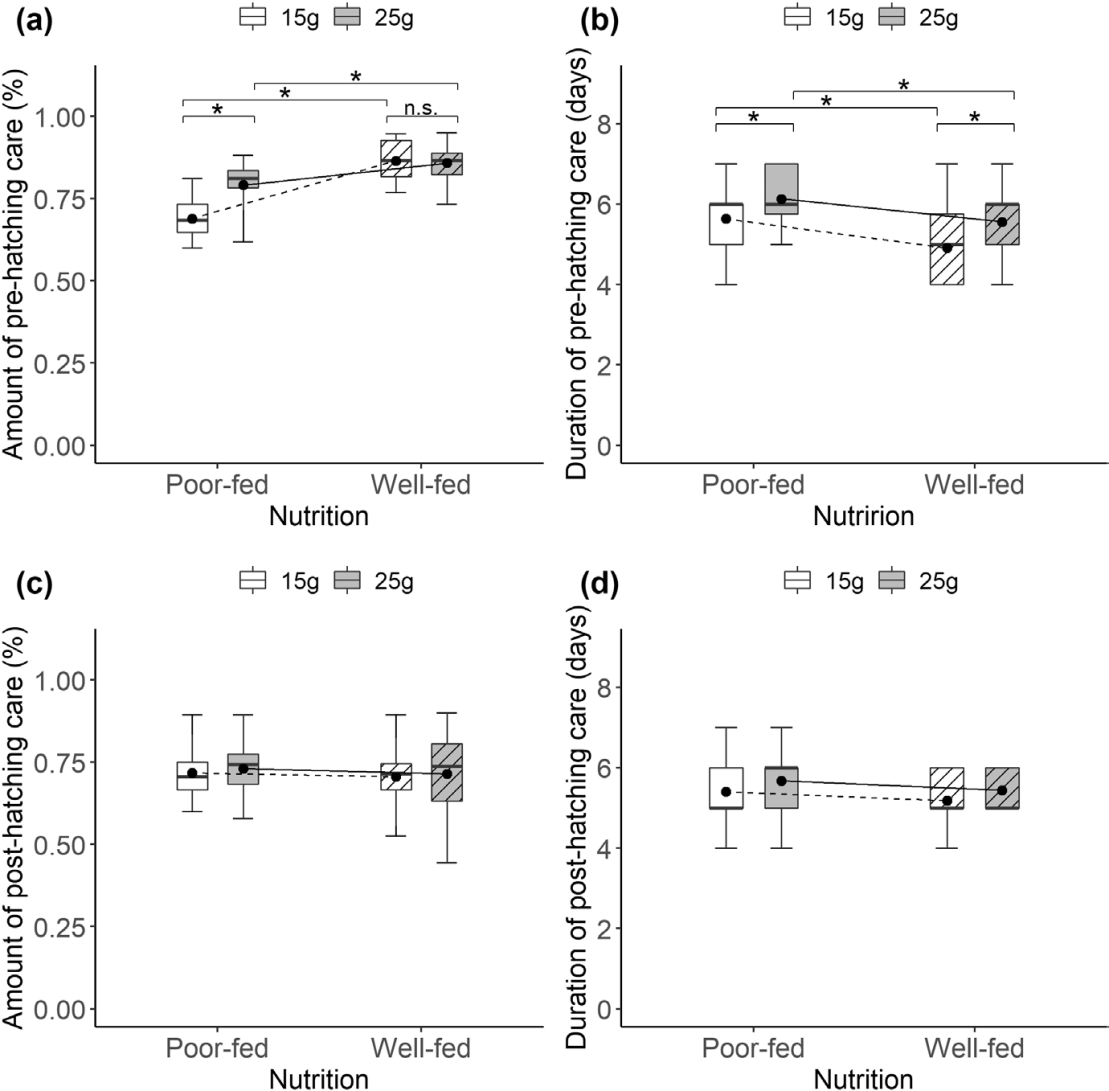
Effects of nutritional status during sexual maturation (poor‐fed vs. well‐fed), carcass size during breeding (15 g vs. 25 g), and the interactive effects on the amount (a) and duration (b) of pre‐hatching care, the amount (c) and duration (d) of post‐hatching care for females during breeding. Black points on each box are mean values. The lines indicate the differences in mean values between different treatments. *Significant (*p* < 0.05); n.s., not significant.

We found that there was a significant interactive effect on the amount of pre‐hatching care for females (Table 2). Poor‐fed females provided more pre‐hatching care when bred on large versus small carcasses (Est = −0.534, *z* = −3.416, *p* = 0.004), whereas well‐fed females provided a similar amount of pre‐hatching care regardless of carcass size (Est = 0.042, *z* = 0.195, *p* = 0.99). In addition, poor‐fed females provided less pre‐hatching care than well‐fed females (Figure 2a). We also compared the duration of pre‐hatching care among different treatments. Poor‐fed females stayed for longer on the carcass than well‐fed females, and females stayed for longer when bred on large versus small carcass (Table 2, Figure 2b). However, we found that females provided a similar amount and duration of post‐hatching care regardless of their nutritional status during sexual maturation and carcass size during breeding (Figure 2c,d).

### 3.3 The Effects of Nutritional Status During Sexual Maturation and Resource Availability During Breeding on the Reproductive Output of Females

**FIGURE 3:**
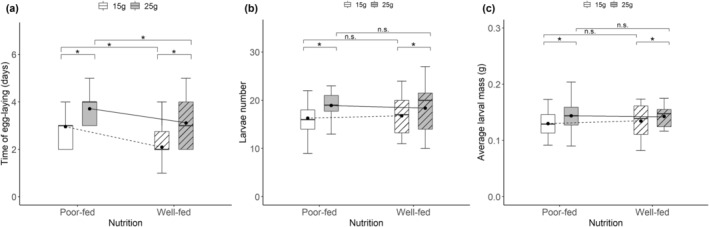
Effects of nutritional status during sexual maturation (poor‐fed vs. well‐fed), carcass size during breeding (15 g vs. 25 g), and the interactive effects on the time of egg‐laying (a), larvae number (b), and average larval mass (c) during breeding. Black points on each box are mean values. The lines indicate the differences in mean values between different treatments. *Significant (*p* < 0.05); n.s., not significant.

**FIGURE 4:**
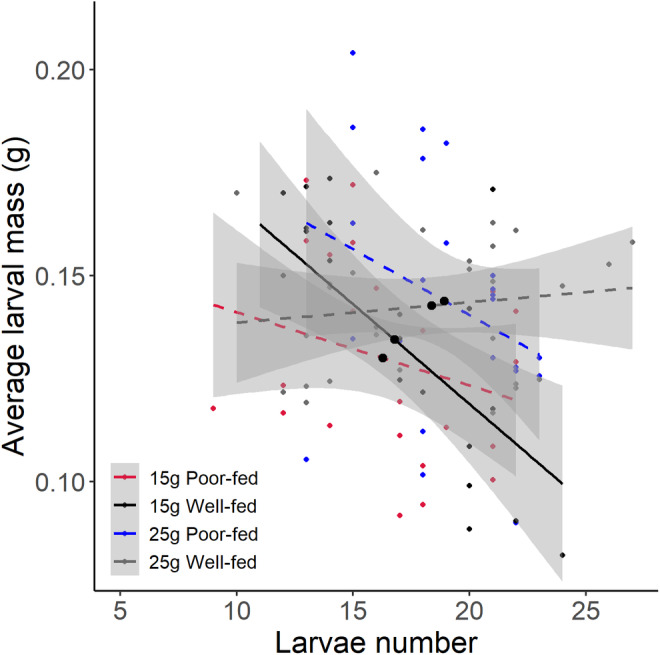
Effects of nutritional status during sexual maturation (poor‐fed vs. well‐fed), and carcass size during breeding (15 g vs. 25 g) on the relationship between larvae number and average larval mass at dispersal. The solid regression lines represent the significant negative relationships; dashed regression lines show nonsignificant relationships (±95% confidence intervals; 15 g poor‐fed, *y* = −0.002*x* + 0.159, *R* ^2^ = 0.03, *p =* 0.20; 15 g well‐fed, *y* = −0.005*x* + 0.216, *R* ^2^ = 0.34, *p =* 0.003; 25 g poor‐fed, *y* = −0.003*x* + 0.204, *R* ^2^ = 0.06, *p =* 0.13; 25 g well‐fed, *y* = 0.001*x* + 0.134, *R* ^2^ < 0.001, *p* = 0.51). Black points on each line are mean values.

We also found that nutritional status during sexual maturation had a significant effect on the egg‐laying time for females but not the larvae number and average larval mass at dispersal (Table 2). Poor‐fed females took longer to start their egg‐laying than well‐fed females (Figure 3a), whereas they produced broods of similar larvae number and average larval mass (Figure 3b,c). In contrast, we found that resource availability had a significant effect on the egg‐laying time, larvae number, and average larval mass (Table 2). Females took longer to start their egg‐laying and produced more and heavier larvae when bred on large versus small carcasses. Finally, only when well‐fed females bred on small carcass, there was a significant negative relationship between larvae number and average larval mass at dispersal (Figure 4).

## 4 Discussion

In this study, we found that poor‐fed females provided more pre‐hatching care when bred on large versus small carcasses, whereas well‐fed females showed no response to carcass size. In addition, variation in nutritional status during sexual maturation affects the resource allocation of burying beetle females only at the pre‐hatching stage. In support of this, poor‐fed females provided less pre‐hatching care and gained more weight than well‐fed females, whereas they provided a similar amount and duration of post‐hatching care. We have no evidence that nutritional status during sexual maturation or resource availability during breeding influences their post‐hatching care. Finally, our findings suggest that poor‐fed females did not suffer reduced offspring number and size. Meanwhile, a large carcass allowed females to produce more and heavier offspring.

### 4.1 The Effects of Nutritional Status During Sexual Maturation and Resource Availability During Breeding on the Weight Change of Females

As predicted, we found that poor‐fed females gained more weight than well‐fed females during the entire breeding. It is unsurprising given that poor‐fed females stayed for longer on the carcass than well‐fed females at the pre‐hatching stage and thus have more chance to feed from the carcass and enhance their energy reserves. This result is consistent with previous studies that burying beetles that were starved prior to breeding consume more resources and gain more weight during breeding (Keppner, Ayasse, and Steiger 2018; Keppner et al. 2023). It reflects that nutritional status during sexual maturation has an important impact on the resource allocation of females, with poor‐fed females allocating more resources into somatic maintenance to compensate for the differences in their nutritional status. Meanwhile, we found that poor‐fed females gained more weight as carcass size increased, whereas well‐fed females gained the same weight regardless of carcass size. It has been suggested that burying beetles have a great requirement for protein during sexual maturation (Al Shareefi and Cotter 2019). Carcasses are superior and protein‐rich resources, and burying beetle parents are known to gain benefits by feeding from the carcass during breeding (Pilakouta, Richardson, and Smiseth 2016; Keppner and Steiger 2021). Therefore, we suggest that poor‐fed females may be deficient in protein and require more resources to enhance their energy reserves, and a large carcass allows them to consume more and gain more weight. However, well‐fed females do not require such a large amount of energy, and thus a small carcass meets their energy requirements.

### 4.2 The Effects of Nutritional Status During Sexual Maturation and Resource Availability During Breeding on the Reproductive Investment of Females

We found that poor‐fed and well‐fed females respond differently to resource availability in terms of the amount of parental care, with poor‐fed females increasing their pre‐hatching care as carcass size increased, whereas well‐fed females provided similar amounts of pre‐hatching care regardless of carcass size. Previous studies have suggested that burying beetle females usually work to their physical limit (Wang et al. 2021, 2022). Therefore, we suggest that well‐fed females may have already worked near their maximum capacity and thus there is no room to increase their pre‐hatching care. This suggestion is also well supported by our result that well‐fed females provided more pre‐hatching care than poor‐fed females whether they were bred on small or large carcasses. We also found that females respond to their own nutritional status during sexual maturation since poor‐fed females stayed for longer on the carcass compared to well‐fed females. This result is unsurprising given that poor‐fed females took longer to complete carcass preparation than well‐fed females, and this is in line with previous studies that starved burying beetles typically perform worse in their adulthood (Hopwood, Moore, and Royle 2013; Richardson, Ross, and Smiseth 2019). This result also confirms that nutritional status during sexual maturation has a long‐term impact on the resource allocation of females in adulthood. In addition, we found that both poor‐fed and well‐fed females stayed for longer as carcass size increased. This is because a larger carcass needs more time to prepare and preserve (Scott 1998; Trumbo 2017), especially in the absence of males. Contrary to our prediction, females provided a similar amount and duration of post‐hatching care regardless of their nutritional status during sexual maturation and resource availability during breeding. One potential explanation for this finding is that poor‐fed females engaged in terminal investment invest relatively heavily in the current reproduction at the post‐hatching stage. Under the terminal investment strategy, individuals with a reduced residual reproductive value should invest more energy in the current reproduction as their future reproductive opportunities decline (Williams 1966). In this study, poor‐fed females were in the suboptimal somatic status and had lower residual reproductive value compared to well‐fed females; they therefore might enhance the investment in the current reproduction at the expense of future reproductive success. In fact, there is already some evidence of terminal investment behavior in this species (Creighton, Heflin, and Belk 2009; Farchmin et al. 2020). Another explanation is that poor‐fed females provided less pre‐hatching care than well‐fed females at the pre‐hatching stage and thus replenished their energy reserves and mitigated the differences in nutritional status. Previous study on other species of burying beetles ( *Nicrophorus orbicollis* ) also demonstrate that burying beetle females quickly recover from starvation by feeding from the carcass (Trumbo and Xhihani 2015). That is, the long‐term effect of nutritional status was offset to some degree, leading to nonsignificant results at the post‐hatching stage. This assumption is supported by our finding that poor‐fed females gained more weight than well‐fed females during the entire breeding. In addition, considering the fact that females typically focus on the post‐hatching stage and work near their physical limitations, we suggest that they have a limited ability to increase their post‐hatching care as carcass size increases, especially after the costly pre‐hatching stage.

### 4.3 The Effects of Nutritional Status During Sexual Maturation and Resource Availability During Breeding on the Reproductive Output of Females

We found that poor‐fed females took longer to start egg‐laying than well‐fed females, which gave them more time to feed from the carcass. There was no difference between poor‐fed and well‐fed females in larvae number and average larval mass at dispersal. It is surprising given that we expected poor‐fed females should allocate more resources to somatic maintenance and thus suffer from reduced future fecundity. Considering that poor‐fed females gain more weight than well‐fed females, we suggest that this egg‐laying delay allowed poor‐fed females to fully compensate for the initial differences in their nutritional status. In addition, we found that both poor‐fed and well‐fed females produced more and larger larvae as carcass size increased. This result is consistent with previous studies (Smiseth et al. 2014; Ratz et al. 2021; Wang et al. 2022), suggesting that a larger carcass allows parents to consume more resources without leading to costs for their offspring. Finally, there was a negative relationship between larvae number and average larval mass at dispersal only when well‐fed females bred on small carcasses, indicating that large carcasses provide enough energy to allocate to both offspring number and size (Wang et al. 2022). In addition, this result was somewhat surprising given that poor‐fed females should have fewer resources for their offspring than well‐fed females and therefore might exhibit a strong trade‐off between larvae number and average larval mass. One potential explanation might be that poor‐fed females engaged in terminal investment and thus mitigated such trade‐offs (Duffield et al. 2017).

## 5 Conclusions

In summary, poor‐fed and well‐fed females vary in their response to resource availability. Poor‐fed females provided more care when bred on large versus small carcasses, whereas well‐fed females tend to work near their maximum capacity and thus show no response to carcass size. In addition, our findings suggest that nutritional status during a key developmental window has an important impact on the resource allocation of burying beetle females at the pre‐hatching stage. In response to nutritional deficiencies, poor‐fed females allocate more resources to somatic maintenance but not reproduction compared to well‐fed females at the early stage of breeding. However, the long‐term effects may diminish with time as females gain benefits by feeding from the carcass. Poor‐fed females finally engaged in terminal investment at the post‐hatching stage because of lower residual reproductive value compared to well‐fed females. This study advances our understanding of resource allocation in burying beetles by demonstrating that nutritional status and resource availability have important interactive consequences for trade‐offs between somatic maintenance and reproduction. The time‐dependent effect of nutritional status in the context of resource availability on resource allocation may help explain why many studies have failed to demonstrate life‐history trade‐offs. However, we only examined their influences over the first breeding attempt; it is unclear what the consequence will be for the lifetime of burying beetles. We thus suggest that future studies should include the lifetime fecundity and lifespan of individuals.

## Author Contributions

**Wenxia Wang:** conceptualization (equal), funding acquisition (lead), writing – original draft (lead). **Guojun Zhou:** data curation (equal), formal analysis (lead), writing – original draft (supporting). **Wei Zhang:** data curation (equal), formal analysis (supporting), writing – review and editing (equal). **Kai Tian:** data curation (equal), visualization (lead), writing – review and editing (equal). **Lunguang Yao:** conceptualization (equal), visualization (supporting), writing – original draft (supporting), writing – review and editing (equal).

## Conflicts of Interest

The authors declare no conflicts of interest.

## Supporting information


Data S1

## Data Availability

Data and R code in this study are available as Data S1.
