# Supplementary material for: Effects of Nutritional Status During Sexual Maturation and Resource Availability on the Resource Allocation of Females in Burying Beetles
Source: Ecol Evol. 2025 Jan 11;15(1):e70808. doi: 10.1002/ece3.70808 (PMC11724369; doi:10.1002/ece3.70808)
Supplement: Supplementary file 1 — Data S1 [file ECE3-15-e70808-s001.zip › R_code.pdf]

Effects of nutritional status during sexual maturation and resource availability on the resource allocation of females in burying beetles

Wenxia Wang, Guojun Zhou, Wei Zhang, Kai Tian, Lunguang Yao

```
## Packages
library(car); library(lme4); library(emmeans)
## Data
a <- read_excel("a.xlsx", col_types = c("text","text",
                                        "numeric","numeric","numeric","numeric","numeric","numeric",
                                        "numeric","numeric","numeric","numeric","numeric","numeric",
                                        "numeric","numeric"))
b <- read_excel("b.xlsx", col_types = c("text","text","text","numeric","numeric","numeric"))
```

Effects of nutritional status and resource availability on the weight change of females

```
#Weight change
WC1 <- lm(weight_change ~ carcass_size*nutrition, data = a)
Anova(WC1)
#interaction: carcass size:nutrition
summary(contrast(emmeans(WC1, ~ carcass_size*nutrition),"pairwise"),
        adjust = "tukey")
```

Effects of nutritional status and resource availability on the pre-hatching care of females

```
#the amount of pre-hatching care
Precare1 <- glmer(cbind(pre_care, pre_observation - pre_care) ~
                          carcass_size*nutrition + (1|id),
                    family = binomial(link = "logit"), data = a)
Anova(Precare1)
#interaction: carcass size:nutrition
summary(contrast(emmeans(Precare1, ~ carcass_size*nutrition),"pairwise"),
        adjust = "tukey")

#the duration of pre-hatching care
PreCareD1 <- (a$pre_care_days)^2 #We improved the model fit by squaring the response variable.
PreCaringDay1 <- glmer(PreCareD1 ~ carcass_size*nutrition+ (1|id),
                          family = poisson(link = "log"), data = a)
Anova(PreCaringDay1)
```

Effects of nutritional status and resource availability on the post-hatching care of females

```
#the amount of post-hatching care
Postcare1 <- glmer(cbind(post_care, post_observation - post_care) ~
                              carcass_size*nutrition*larvae_number + (1|id),
                     family = binomial(link = "logit"), data = a)
Anova(Postcare1)
#The treatment-covariate interactions were not significant and thus excluded in the final model.

Postcare2 <- glmer(cbind(post_care, post_observation - post_care) ~
                              carcass_size*nutrition + larvae_number + (1|id),
                     family = binomial(link = "logit"), data = a)
Anova(Postcare2)

#the duration of post-hatching care
PostCareD <- (a$post_care_days)^2#We improved the model fit by squaring the response variable.
PostCaringDay1 <- glmer(PostCareD ~ carcass_size*nutrition + (1|id),
                        family = poisson(link = "log"), data = a)
Anova(PostCaringDay1)
```

Effects of nutritional status and resource availability on the offspring performances

```
#the time of Egg-laying
EggLaying <- (a$egg_laying_time)^2#We improved the model fit by squaring the response variable.
EggLayingDay1 <- glmer(EggLaying ~ carcass_size*nutrition+ (1|id),
                       family = poisson(link = "log"), data = a)
Anova(EggLayingDay1)

#offspring number and size
LarvaeNumber1 <- glm(larvae_number ~ carcass_size*nutrition,
                     family = poisson(link = "log"), data = a)
Anova(LarvaeNumber1)
AverageMass1 <- lm(average_larvae_mass ~ carcass_size*nutrition*larvae_number, data = a)
Anova(AverageMass1)
#The treatment-covariate interactions were highly significant,
#indicating that the homogeneity of the slopes assumption was violated.
AverageMass2 <- lm(average_larvae_mass ~ carcass_size*nutrition, data = a)
Anova(AverageMass2)
#We reported the results of the model of average larval mass excluding larvae number as a covariate.
```

The differences in body size and body weight of parents among different treatments

```
#the pronotum width and initial body weight of parents post-eclosion
summary(aov(body_size ~ carcass_size*nutrition*sex, data = b))
summary(aov(weight_post_eclosion ~ carcass_size*nutrition*sex, data = b))
#the body weight of poor-fed and well-fed females after nutritional treatments
summary(aov(weight_at_breeding ~ carcass_size*nutrition, data = b))
```
